# Selective *selC*-Independent Selenocysteine Incorporation into Formate Dehydrogenases

**DOI:** 10.1371/journal.pone.0061913

**Published:** 2013-04-25

**Authors:** Michael Zorn, Christian H. Ihling, Ralph Golbik, R. Gary Sawers, Andrea Sinz

**Affiliations:** 1 Department of Pharmaceutical Chemistry & Bioanalytics, Institute of Pharmacy, Martin-Luther University Halle-Wittenberg, Halle (Saale), Germany; 2 Institute of Microbiology, Martin-Luther University of Halle-Wittenberg, Halle (Saale), Germany; 3 Institute of Biochemistry, Martin-Luther University of Halle-Wittenberg, Halle (Saale), Germany; National Institutes of Health, United States of America

## Abstract

The formate dehydrogenases (Fdh) Fdh-O, Fdh-N, and Fdh-H, are the only proteins in *Escherichia coli* that incorporate selenocysteine at a specific position by decoding a UGA codon. However, an excess of selenium can lead to toxicity through misincorporation of selenocysteine into proteins. To determine whether selenocysteine substitutes for cysteine, we grew *Escherichia coli* in the presence of excess sodium selenite. The respiratory Fdh-N and Fdh-O enzymes, along with nitrate reductase (Nar) were co-purified from wild type strain MC4100 after anaerobic growth with nitrate and either 2 µM or 100 µM selenite. Mass spectrometric analysis of the catalytic subunits of both Fdhs identified the UGA-specified selenocysteine residue and revealed incorporation of additional, ‘non-specific’ selenocysteinyl residues, which always replaced particular cysteinyl residues. Although variable, their incorporation was not random and was independent of the selenite concentration used. Notably, these cysteines are likely to be non-essential for catalysis and they do not coordinate the iron-sulfur cluster. The remaining cysteinyl residues that could be identified were never substituted by selenocysteine. Selenomethionine was never observed in our analyses. Non-random substitution of particular cysteinyl residues was also noted in the electron-transferring subunit of both Fdhs as well as in the subunits of the Nar enzyme. Nar isolated from an *E. coli selC* mutant also showed a similar selenocysteine incorporation pattern to the wild-type indicating that non-specific selenocysteine incorporation was independent of the specific selenocysteine pathway. Thus, selenide replaces sulfide in the biosynthesis of cysteine and misacylated selenocysteyl-tRNA^Cys^ decodes either UGU or UGC codons, which usually specify cysteine. Nevertheless, not every UGU or UGC codon was decoded as selenocysteine. Together, our results suggest that a degree of misincorporation of selenocysteine into enzymes through replacement of particular, non-essential cysteines, is tolerated and this might act as a buffering system to cope with excessive intracellular selenium.

## Introduction

The 21^st^ amino acid is selenocysteine and it is co-translationally incorporated at a specific position in the amino acid sequence of selenoproteins such as the formate dehydrogenases (Fdh) of *Escherichia coli* [1], [2]. Specific incorporation of selenocysteine is directed by an in-frame UGA codon in the mRNA encoding these enzymes. In order for the decoding to outcompete translational termination successfully four *sel* gene products are required whose functions include the biosynthesis and incorporation of selenocysteine [2]. The key gene is *selC*, which encodes tRNA^Sec^ whose anticodon recognizes a particular UGA codon and decodes it when aminoacylated with selenocysteine. Mutations in any of the *sel* genes prevent translation of the UGA codon resulting in premature termination of translation and a failure to synthesize active Fdh [3].

As well as being an essential trace element excess selenium can be toxic for cells. This is mainly due to the high reactivity of the selenol group, e.g. of selenocysteine, which is strongly nucleophilic [4]. With a pK_a_ of 5.2 compared with 8.3 for the thiol, selenol groups are therefore essentially ionized at neutral pH. The advantage of the selenol over thiol in catalysis is exemplified by a Fdh-H Sec→Cys variant of *E. coli*, which is approximately 300-fold less catalytically active compared with the wild-type selenocysteine-containing enzyme [5]. The disadvantage of excessive and unspecific selenol groups inside cells is that they disrupt numerous cellular processes. The limited studies performed on selenium toxicity in *E. coli* strongly suggest that selenium is incorporated non-specifically into proteins as selenocysteine, presumably by replacing cysteines [6], [7]. Despite early reports suggesting that selenomethionine could also be synthesized and incorporated in *E. coli* [8], later studies with mutants defective in cysteine and methionine biosynthesis [7] indicated that selenocysteine is likely the form in which selenium is non-specifically incorporated into proteins. Moreover, the fact that the *O*-acetylserine synthetases of *E. coli* can accept selenide as a substrate [9], coupled with the effective aminoacylation of cysteinyl-tRNA by selenocysteine [6], [10] supports this proposal. One of the aims of this study, therefore, was to demonstrate using mass spectrometry whether selenocysteine indeed replaces cysteine in *E. coli* proteins.

The Fdh-N and Fdh-O selenoenzymes of *E. coli* have an energy-conserving function [11]. Limited information is available about Fdh-O, although it has formate oxidase activity during aerobic growth [12], [13] and it is also synthesized during nitrate respiration [14], [15]. Considerably more is known about the nitrate-inducible Fdh-N enzyme, which forms a respiratory chain with nitrate reductase (Nar) during anaerobic nitrate respiration [11], [16], [17]. Structural analyses of both the Fdh-N and Nar enzymes [18], [19], [20] have helped provide a molecular framework explaining Mitchell’s redox-loop theory of chemiosmosis [21], [22].

The highly similar Fdh-N and Fdh-O enzymes belong to the molybdopterin-containing oxidoreductase family [18], [23]. Both enzymes are composed of three subunits. The α-subunit (FdnG/FdoG) contains the catalytically active selenocysteine, an iron-sulfur [4Fe-4S] cluster as well as two bis-MGD cofactors [16], [17], [18]. The electron-transferring ß-subunit (FdnH/FdoH) has four [4Fe-4S] clusters and the γ-subunit (FdnI/FdoI) is an integral membrane protein that anchors the other two subunits to the periplasmic side of the membrane. The γ-subunit has two heme *b* groups and a menaquinone binding site [18]. Nar has a similar overall architecture to the Fdh enzymes, but has its active site oriented towards the cytoplasm and lacks a selenocysteine [19], [20].

The Fdh-N and Fdh-O isoenzymes present an ideal system to study the non-specific incorporation of selenium-containing amino acids into proteins, because they both have a single selenocysteinyl residue that is incorporated specifically and thus acts as an internal standard. Moreover, they have a significant number of cysteinyl residues in both their catalytic and electron-transferring subunits and the Fdh-N enzyme is known to be an abundant membrane protein in nitrate-grown *E. coli* cells [16]. Using selenite as a source of selenium [24], we exposed anaerobic, nitrate-grown *E. coli* cells to different concentrations of selenium and subsequently co-purified the Fdh and Nar enzymes. In-depth mass spectrometric analyses provided convincing evidence that selenocysteine residues only replace particular cysteines in all three enzymes. The surprising finding that cysteines are not replaced randomly by selenocysteine upon selenium excess suggests there is a mechanism in place to ensure catalytically and structurally important cysteines are not substituted.

## Materials and Methods

### Materials and Reagents

Ammonium bicarbonate (99%), 3-(*N*-morpholino)propanesulfonic acid (MOPS), and formic acid (mass spectrometry grade) were purchased from Sigma (Taufkirchen, Germany). Trypsin gold (mass spectrometry grade) was obtained from Promega (Madison, WI, USA). Acetonitrile (LiChroSolv® and for Mass Spectrometry) was obtained from Merck (Darmstadt, Germany) and JT Baker (Griesheim, Germany). Acetic acid, *N*-dodecyl-β-D-maltoside (DDM), peptone, Triton X-100, and yeast extract were obtained from Carl Roth GmbH & Co. KG (Karlsruhe, Germany). Protease inhibitor cocktail Complete (EDTA-free) was obtained from Roche (Mannheim, Germany), phenylmethylsulfonylfluoride (PMSF) was obtained from Molekula GmbH (München, Germany).

### Growth Conditions

Fermentation of *E. coli* MC4100 and its *selC* derivative FM460 [13] was carried out using a bioreactor (Biostat ED Reactor) with the central process management system MCFSwin. First, 5 L of 1% (w/v) peptone, 0.5% (w/v) yeast extract were autoclaved for 45 min at 121°C in the reactor. The medium was subsequently cooled down to room temperature and the pH was adjusted to 7.5 using 1 L of sterile 1 M potassium hydrogen phosphate buffer. Subsequently, the medium was supplemented with 0.8% (w/v) glucose, 100 µM sodium molybdate, and 100 µM sodium selenite. The automatic control system allowed continuous and steady aeration with molecular oxygen. The oxygen partial pressure was set to a minimum of 30%. Cells from a 2-L preculture were harvested by centrifugation, suspended in 100 ml of fresh sterile medium, serving for the inoculation of the bioreactor. Fermentation was performed at 37°C and at an oxygen saturation of minimally of 30%. Titration of the pH to 7.5 was automated by supply of either 10% (v/v) phosphoric acid or 10% (w/v) potassium hydroxide. Bacterial growth was promoted by continuous addition of a feeding solution comprising 7% (w/v) peptone, 3.5% yeast (w/v), 0.8% (w/v) glucose, 100 µM sodium molybdate and 100 µM sodium selenite. After 22 h of growth, the oxygen partial pressure was set to 0% followed by the addition of 100 mM potassium nitrate. Cells were cultivated for an additional 2.5 hours before harvesting by centrifugation. Finally, cells were shock-frozen in liquid nitrogen and stored at −20°C. Alternatively, *E. coli* wild type MC4100 or the isogenic *selC* mutant FM460 were cultivated in 15-L batch cultures at 22°C. Cells were grown anaerobically in TGYEP medium [25] supplemented with 100 mM potassium nitrate, 2 µM sodium selenite and 2 µM sodium molybdate.

### Enrichment of Enzymes Involved in Formate-Dependent Nitrate Respiration

50 g wet-weight of cells were resuspended in 150 ml of 50 mM MOPS, 1 µM DNase I, 2 mM PMSF, pH 7.5 and disrupted by three passages through a French Press. Subsequent steps were carried out at 0–4°C unless stated otherwise. The lysate was centrifuged at 28,000×g for 30 min. The cell-free supernatant was then centrifuged at 40,000 rpm for 2 hrs using a Beckman Coulter ultracentrifuge (Beckman Coulter, Optima L Series; Type 45 Ti Rotor). The membrane fraction was resuspended in 50 mM MOPS, 1% (w/v) DDM, pH 7.5, and the protein concentration was adjusted to 5 mg/ml. Membrane proteins were solubilized by gently shaking the mixture at 8°C for 1 hr. Insoluble membrane material was separated from solubilized proteins by an additional centrifugation step at 40,000 rpm for 1 hr. Afterwards, ammonium sulfate was added slowly to the supernatant to give a 50% saturated solution. The suspension was stirred for 1 hr and the precipitate was removed by centrifugation at 28,000×*g* for 30 min. The supernatant was used to generate 60, 70 and 80% ammonium sulfate protein fractions in consecutive precipitation and centrifugation steps. The precipitate from the 70% ammonium sulfate fractionation step was recovered in 5 ml 50 mM MOPS, 1 M ammonium sulfate, pH 7.5. Protein purification was performed using an ÄKTA FPLC system (ÄKTA FPLC, GE Healthcare). The mixture was applied to a butyl sepharose column (4.9 ml; GE Healthcare) pre-equilibrated with at least 10 column volumes of equilibration buffer (50 mM MOPS, 1 M ammonium sulfate, pH 7.5). The column was then developed using a gradient from 1 M to 0 M ammonium sulfate (in 50 mM MOPS, pH 7.5) over 10 column volumes. Finally, proteins were eluted with MilliQ-water containing 0.5% (w/v) Triton X-100. All fractions showing formate dehydrogenase (Fdh) activity were pooled and concentrated to a volume of 2 ml using an Amicon cell with a 10 kDa cutoff filter (Millipore). Simultaneously, buffer was exchanged to 50 mM MOPS, 1 M NaCl, 0.5% (w/v) Triton X-100, pH 7.5. The concentrated protein solution was applied to a Sephacryl S400 size-exclusion column (320 ml; GE Healthcare) pre-equilibrated with 2 column volumes of buffer (50 mM MOPS, 1 M NaCl, 0.5% (w/v) Triton X-100, pH 7.5), and the column was developed using the same buffer. All fractions showing Fdh activity were pooled, the solution was concentrated to a volume of 2 ml, and the buffer was exchanged to 50 mM MOPS, pH 7.5. This sample was applied to an anion exchange column (MonoQ 5/50 GL, Amersham Biosciences) equilibrated with 50 mM MOPS, pH 7.5, 0.1% (w/v) Triton X-100). The column was washed with equilibration buffer before proteins were eluted with a linear gradient from 0–100% using 50 mM MOPS, 1 M NaCl, 0.1% (w/v) Triton X-100, pH 7.5. Fdh-containing fractions were concentrated, rapidly frozen in liquid nitrogen and stored at −20°C until use.

### Enzyme Assay

Fdh-containing fractions were identified by the formate-dependent reduction of 2,4-dichlorophenolindophenol (DCPIP) in the presence of phenazine methosulfate at 37°C as described [16]. The reaction mixture (1 ml) contained 50 mM MOPS, 80 mM sodium formate, 260 µM PMS, 120 µM DCPIP, pH 7.5. The reduction of DCPIP was initiated by adding 5 to 50 µl of protein solution.

### SDS-PAGE and In-Gel Digestion

SDS-PAGE (12% w/v acrylamide) under reducing conditions was used to separate polypeptides. After staining with Coomassie Brilliant Blue, gel pieces were excised and *in-gel* digested with trypsin (Promega) following a standard protocol [26].

### Mass Spectrometry

*In-gel*-digested peptides were analyzed by LC/MS on an UltiMate Nano-HPLC system (LC Packings/Dionex) coupled to an LTQ-Orbitrap XL mass spectrometer (ThermoFisher Scientific) equipped with a nano-electrospray ionization source (Proxeon). The samples were loaded onto a trapping column (Acclaim PepMap C18, 100 µm×20 mm, 5 µm, 100Å, LC Packings) and washed for 15 min with 0.1% (w/v) TFA at a flow rate of 20 µl/min. Trapped peptides were eluted using a separation column (Acclaim PepMap C18, 75 µm×250 mm, 3 µm, 100Å, LC Packings) that had been equilibrated with 100% A (5% (v/v) acetonitrile, 0.1% (v/v) formic acid). Peptides were separated with linear gradients from 0–40% B (80% (v/v) acetonitrile, 0.08% (v/v) formic acid) over 90 min. The column was kept at 30°C and the flow rate was 300 nl/min. *Online* MS data were collected in data-dependent MS/MS mode during the complete gradient elution: Each high-resolution full scan (*m/z* 300 to 2000, resolution 60,000) in the orbitrap analyzer was followed by five product ion scans (collision-induced dissociation (CID)-MS/MS) in the linear ion trap for the five most intense signals of the full scan mass spectrum (isolation window 2.5 Th). Dynamic exclusion (repeat count was 3, exclusion duration 180 s) was enabled to allow detection of less abundant ions. Data analysis was performed using the Proteome Discoverer 1.2 (Thermo Fisher Scientific), MS/MS data of precursor ions (*m/z* 500–5000) were searched against the SwissProt Database (version 11/2010, taxonomy *E. coli*, 22,729 entries) using Mascot (version 2.2, Matrixscience). Mass accuracy was set to 3 ppm and 0.8 Da for precursor and fragment ions, respectively. Carbamidomethylation of cysteine and selenocysteine, substitution of cysteine for dehydroalanine and selenocysteine, and oxidation of methionine were set as potential modifications, and up to three missed cleavages of trypsin were allowed.

## Results

### Purification of Fdh-N and Fdh-O

**Figure 1:**
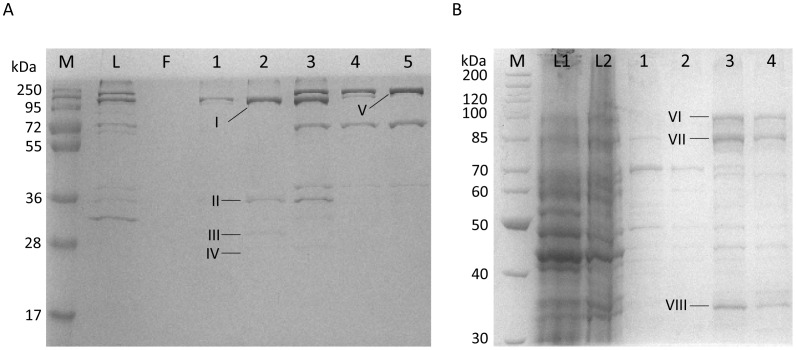
SDS-PAGE of the purification step by anion exchange chromatography (MonoQ 5/50 GL column). (A) Protein purification from MC4100 cells cultivated in TGYEP medium supplemented with 2 µM sodium selenite and 2 µM sodium molybdate; 12% (w/v) resolving gel; M: molecular weight marker; L: sample applied to the column; F, column flow-through; 1–5: elution fractions. (B) Protein purification from MC4100 cells cultivated in TGYEP medium supplemented with 100 µM sodium selenite and 100 µM sodium molybdate; 8% (w/v) resolving gel; M: molecular weight marker; L1: sample loaded on the size exclusion column; L2:sample loaded on the anion exchange column; 1–4: elution fractions from the anion-exchange column. Polypeptides labeled I though VIII were those identified by mass spectrometry and are listed in Table 1.

The two formate dehydrogenase isoenzymes Fdh-N and Fdh-O were simultaneously purified under native conditions from cells that were grown anaerobically in rich medium containing sodium nitrate and supplemented with either 2 µM (batch culture; Figure 1A) or 100 µM (fermenter; Figure 1B) sodium selenite. Briefly, membrane fractions derived from these cells were solubilized, precipitated with ammonium sulfate, and the fraction obtained with 70% ammonium sulfate was subjected to three consecutive chromatographic steps comprising hydrophobic interaction, size-exclusion and anion exchange chromatography steps. The last purification step resulting in Fdh-N and Fdh-O is shown in Figure 1. The native Fdhs co-purified but we noted occasionally the presence of minor contaminants associated with the purified fraction. The cysteine concentration in the rich medium was initially approximately 1 mM [27]. Optimal selenium incorporation has been observed when selenium is provided in the form of selenite at a concentration of 100 nM and when cysteine is present as a sulfur source at 80 µM, i.e., in an almost thousand-fold excess over sodium selenite [2]. Concentrations above 1 µM selenite have been shown to result in non-specific selenium incorporation [28]. Two excess concentrations of sodium selenite were used in our experiments. Selenite was added at a concentration of 2 µM (approximately 500-fold molar excess of cysteine in the medium) and at a concentration of 100 µM. Both concentrations allowed an effective assessment of the consequences of an over-abundance of selenite relative to cysteine on specific and non-specific selenocysteine incorporation into the polypeptides of the Fdhs.

Fdh-N- and Fdh-O-containing fractions were subjected to SDS-PAGE separation and the polypeptides were excised from the gel and analyzed by mass spectrometry. SDS-PAGE analysis (Figure 1) suggested the presence of all three subunits (G, H, and I) of both enzymes had been isolated, and indeed, all three subunits were identified for both Fdh-N and Fdh-O using mass spectrometry (Table 1). NarG was successfully separated from the Fdh polypeptides at the final purification step (see also [16]); however, OmpA was occasionally observed to comigrate with the Fdhs after purification from cells grown with 100 µM selenite (Figure 1B, Table 1). FdoI appeared as a rather faint band on the SDS gel (band IV, Figure 1A), presumably because of its hydrophobic properties [11], [16]. Nevertheless, we were also able to unambiguously identify this subunit. Based on both the total coverage and the amounts of the three polypeptides of each enzyme identified, it is estimated that Fdh-O is minimally as abundant as Fdh-N in nitrate-grown cells.

**Table 1:** Proteins obtained in fractions 2, 3, and 5 (see Figs. 1A and B) from anion exchange chromatography that were identified by nano-ESI-LTQ-Orbitrap-MS/MS analysis; NCBI entry numbers are given.

Gel band(see Fig. 1)	Protein	NCBI entry	# of matchedpeptides	Mascot score
I	FdnG	GI:20150976	64	9352
	FdoG	GI:85676165	52	6298
II	FdnH	GI:1742408	18	1179
	FdoH	GI:85676166	15	521
III	FdnI	GI:1742409	2	55
IV	FdoI	GI:85676167	3	173
V	NarG	GI:4062800	73	7963
VI	FdoG	GI:8567615	48	2727
	FdnG	GI:20150976	28	836
VII	FdoG	GI:8567615	44	3045
	FdnG	GI:20150976	21	781
VIII	FdoH	GI:85676166	19	794
	FdnH	GI:1742408	8	235
	OmpA	GI:1651465	11	431

### Identification of Selenopeptides

The deduced amino acid sequences of the catalytic subunit of Fdh-O and Fdh-N each have one selenocysteinyl residue (at amino acid position 196) and either 15 or 14 cysteinyl residues in the case of FdoG and FdnG, respectively [14], [17]. The electron-transferring H subunit has 16 (FdoH) or 17 (FdnH) cysteinyl residues, while the membrane anchor subunit FdoI/FdnI has two cysteines in each case. After having confirmed the identity of the purified Fdhs, we next investigated whether additional selenocysteinyl or selenomethionyl residues were present in any of the polypeptides. Selenomethionine was never observed in any polypeptide during our analyses, confirming earlier proposals, which strongly suggested that non-specific selenium incorporation was mainly in the form of selenocysteine [2], [7].

**Figure 2:**
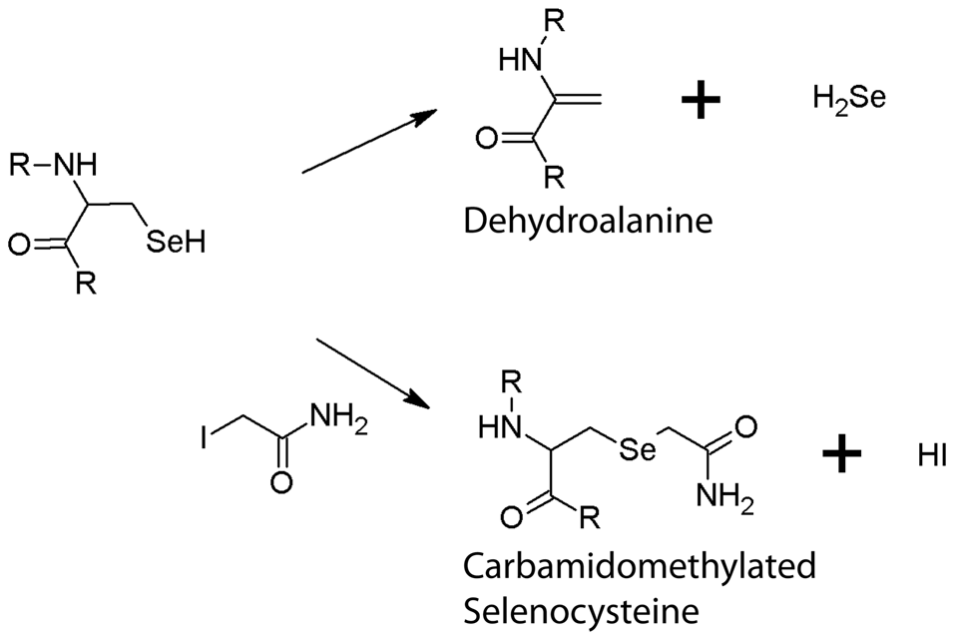
Conversion of selenocysteine into dehydroalanine (upper path) and carbamidomethylated selenocysteine by iodoacetamide (lower path).

**Figure 3:**
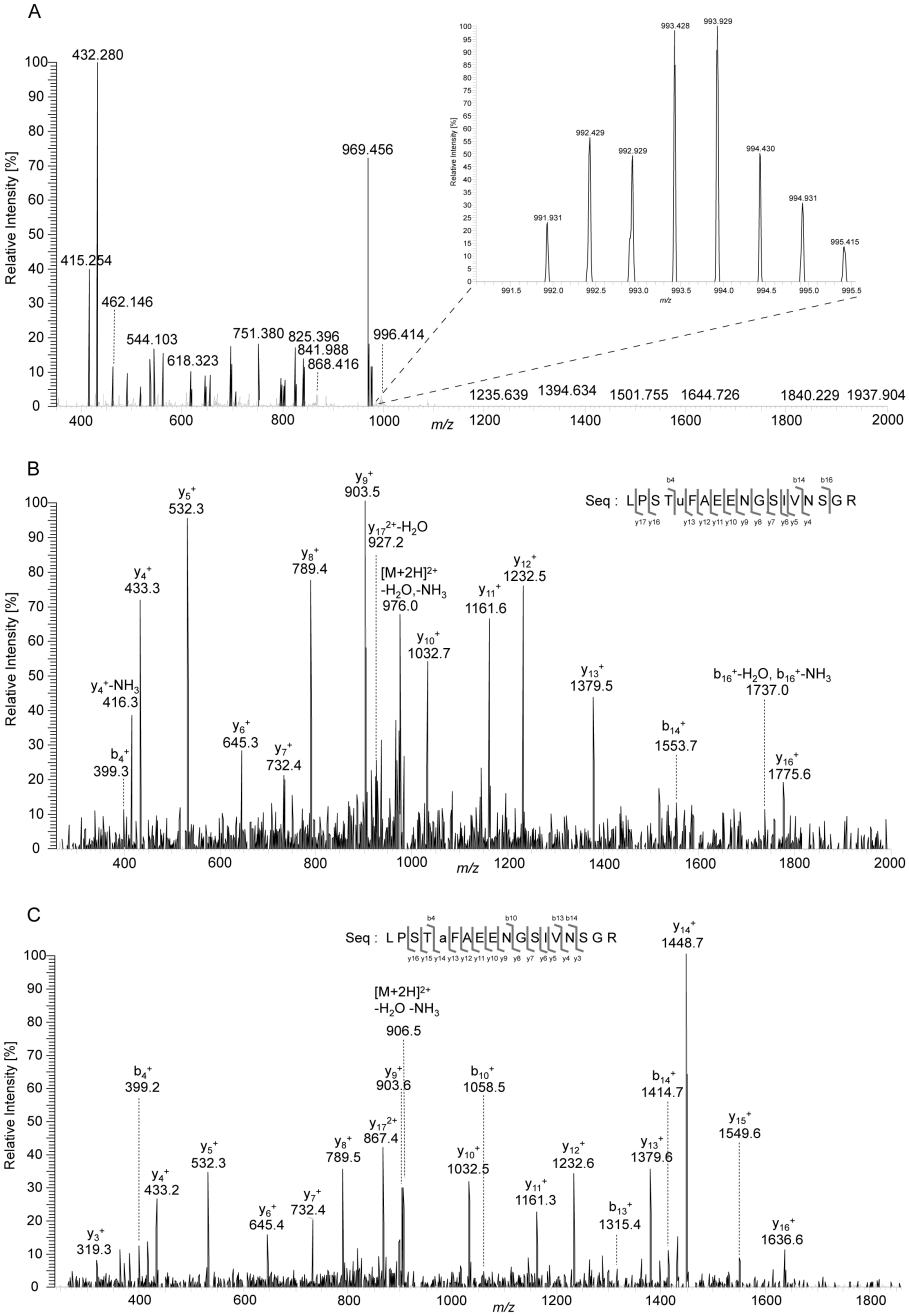
(A) Nano-ESI-LTQ-Orbitrap-MS data. The signal of the selenopeptide from FdoG at *m/z* 993.4281 matches the expected mass with a deviation of 1.1 ppm. In the inset the signal is shown enlarged, exhibiting the characteristic isotope pattern of a selenopeptide. (B, C) Nano-ESI-LTQ-Orbitrap-MS/MS data of peptides LPSTu_618_FAEENGSIVNSGR (B, *m/z* 993.4281) and LPSTa_618_FAEENGSIVNSGR (C, *m/z* 923.9503). Precursor ions were selected, fragmented, and analyzed in the linear ion trap (LTQ). MS/MS data unambiguously identify carbamidomethylated selenocysteine (u) and dehydroalanine (a) at position 618.

Mass spectrometric confirmation of selenocysteine is a rather challenging task as selenium loss occurs during sample preparation leading to the conversion of selenocysteine to dehydroalanine, which results in a mass loss of 82 u [29] (Figure 2). Selenium-containing peptides are readily recognized in mass spectra based on their characteristic isotope patterns as selenium naturally occurs in six isotopes: ^74^Se (0.9%), ^76^Se (9.0%), ^77^Se (7.6%), ^78^Se (23.6%), ^80^Se (49.7%), and ^82^Se (9.2%). For the MS analyses two complementary approaches were chosen: The proteins isolated from cells grown in the presence of 2 µM selenite were reduced and treated with iodoacetamide, which resulted in the carbamidomethylation of all accessible cysteines and selenocysteines (mass increase of 57 u; Figure 2). Based on MS/MS experiments, we unambiguously identified both dehydroalanines as well as carbamidomethylated selenocysteines in the respective proteins (Figure 3, Figure S1). In the proteins isolated from cells grown in the presence of 100 µM selenite, on the other hand, no alkylation step was included to test an alternative MS identification strategy of selenocysteines. This resulted in the exclusive identification of dehydroalanines, which are indicative of the presence of selenocysteines in the polypeptides (Table 2). Selenopeptides originating from Fdh-O and Fdh-N were the main focus of this study, but we also made use of the fact that Nar, and in the proteins isolated from the growth with 100 µM selenite OmpA, co-purified with the Fdhs and these acted as further controls for selenocysteine incorporation. In most cases, we were able to unambiguously assign the selenium-containing peptides to one single protein. Only in three cases did sequence identities between FdoG/FdnG, FdoH/FdnH and NarG/NarZ preclude an unambiguous assignment of the respective selenopeptides (Table 2).

**Table 2:** Summary of peptides in *E. coli* MC4100, in which cysteines were found to be substituted by selenocysteine and dehydroalanine by ESI-LTQ-Orbitrap-MS/MS.

Protein ID	Position and type of substitution^*1^	Precursor ion mass			Δm [ppm]	Cultivation conditions*^2^	
		*m/z* [M+H]^+^_exp_	Charge state	*m/z* [M+H]^+^_theor_		100 µM	2 µM
FdoG	LPST**u_618_**FAEENGSIVNSGR	993.4281	+2	1985.8489	1.12	+	
	LPST**a_618_**FAEENGSIVNSGR	923.9503	+2	1846.8934	0.19	+	+
	V**u_391_**EYIAETSAHDK	785.8182	+2	1570.6292	0.30	+	
	V**a_391_**EYIAETSAHDK	716.3411	+2	1431.6749	0.09	+	+
	WLSTGML**a_165_**ASASSNETGYLTQK	1165.5532	+2	2330.0991	0.92		+
	VNGYI**a_554_**QGFNPVASFPNK	960.9849	+2	1920.9627	1.22		+
FdnG	WLSTGML**a_165_**ASGASNETGMLTQK	1134.5362	+2	2268.0652	0.71		+
	VTGYF**a_554_**QGFNPVASFPDK	971.9710	+2	1942.9347	0.66		+
	LPST**a_618_**FAEEDGSIANSGR	910.4268	+2	1819.8463	0.29		+
	V**a_391_**EVLASTSAPDR	657.3382	+2	1313.6692	0.30		+
FdoG or	V**u_196_**HGPTVASLAPTFGR	859.4000	+2	1717.7934	0.61	+	
FdnG	V**a_196_**HGPTVASLAPTFGR	789.9236	+2	1578.8408	0.82	+	+
	YTPDVVENI**u_380_**GTPK	820.8578	+2	1640.7084	0.87	+	
	YTPDVVENI**a_380_**GTPK	751.3800	+2	1501.7528	0.34	+	+
FdoH	LIDVTT**a_39_**IGcK	594.8177	+2	1188.6283	0.87	+	
	LIDVTTcIG**u_42_**K	664.2949	+2	1327.5826	0.26	+	
	LIDVTTcIG**a_42_**K	566.3073	+2	1131.6073	0.43	+	+
FdnH	DEVGH**a_62_**VGVYDNPADLSAK	977.9615	+2	1954.9158	0.83		+
FdoH or	A**u_45_**QVAcSEWNDIR	828.8214	+2	1656.6356	1.10	+	
FdnH	AcQVA**a_49_**SEWNDIR	759.3435	+2	1517.6798	0.25	+	
	cTL**a_160_**VDR	416.7028	+2	832.3983	0.15	+	
	T**a_179_**PTGAIHFGTK	599.8140	+2	1198.6208	0.62	+	+
OmpA	GMGESNPVTGNT**u_311_**DNVK	922.3557	+2	1843.7041	0.56	+	
	GMGESNPVTGNT**a_311_**DNVK	852.8782	+2	1704.7491	0.17	+	+
	AALID**a_323_**LAPDR	562.3084	+2	1123.6096	0.89		+
NarG	GLNDVN**a_493_**ATSYDDVK	790.3660	+2	1579.7248	0.84		+
	KFSEV**a_838_**VGHLGK	635.3511	+2	1269.6950	0.01		+
	FSEV**a_838_**VGHLGK	381.2044	+2	1141.5987	1.12		+
	L**a_292_**DLWLAPK	512.7946	+2	1024.5821	0.52		+
NarH	IEAGQPTVCSET**a_263_**VGR	808.3910	+2	1615.7748	0.18		+
NarG or	KGE**a_875_**DLIPGK	513.2845	+2	1025.5618	0.82		+
NarZ	GE**a_875_**DLIPGK	449.2371	+2	897.4669	0.80		+

Charge states of precursor ions that were selected for MS/MS analysis are indicated. *^1^: C: cysteine; c: carbamidomethylated cysteine; u: carbamidomethylated selenocysteine; a: dehydroalanine; selenocysteine at position 196 is the naturally occurring amino acid. *^2 ^Two complementary protein purifications were performed from MC4100 cells cultivated with different concentrations of the trace elements selenium and molybdenum. Cultures were supplemented with 2 µM or 100 µM of sodium selenite and sodium molybdate, respectively.

**Figure 4:**
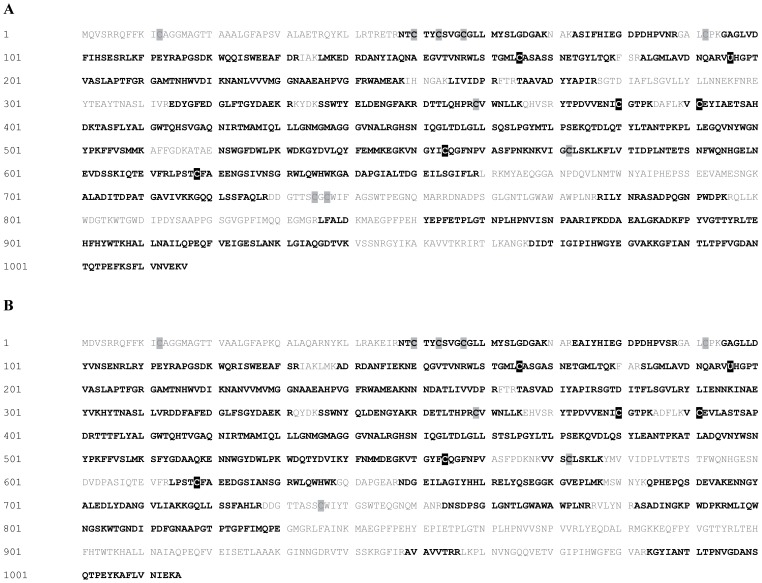
Amino acid sequences of (A) FdoG and (B) FdnG. Peptides identified by MS/MS analysis are shown in bold, while sequences that were not covered by MS analysis are shown in grey. Cysteines are shown in grey boxes, selenocysteines and dehydroalanines are shown in black boxes. Sec-196 (U) is the specific selenocysteine amino acid inserted by decoding of the UGA codon. (A) Sequence coverage of FdoG is 70.5%, selenocysteine substitutions were identified at positions 165, 380, 391, 554, and 618. (B) Sequence coverage of FdnG is 77.0%, selenocysteine substitutions were identified at positions 165, 380, 391, 554, and 618.

Selenocysteine was specifically incorporated by tRNA^Sec^ at position 196 (UGA codon) in both FdoG and FdnG polypeptides [14], [17] and we never identified a cysteinyl residue at this position. This finding serves as additional confirmation for the validity of our MS approach. Sequence coverage of the catalytic FdnG and FdoG subunits ranged between 60% and 77%, while that of the electron-transferring H subunit ranged between 50% and 60%. The sequence coverage of the membrane-anchoring FdnI/FdoI subunits was only 18%. Therefore, it was not possible to determine whether selenocysteine was incorporated at the position of cysteine in any of those portions of the polypeptides not covered by our MS analysis. Remarkably, five cysteines were replaced by selenocysteine and these were at identical positions in the catalytic subunit of both Fdh-N and Fdh-O, regardless of whether 2 µM or 100 µM sodium selenite was used during cultivation (Fig. 4 and Table 2). It was noted that there was variability in the frequency of cysteine for selenosysteine exchange at some positions, while this was not observed for amino acid positions 165 in both FdoG and FdnG and 554 in FdnG, which were always identified as selenocysteine (Table 3). While Cys165 was coded by UGU, Cys554 was coded by UGC, indicating that there was no apparent bias toward decoding either of the Cys codons with selenocysteinyl-tRNA^Cys^.

**Table 3:** Overview of peptides containing cysteines, selenocysteines, and dehydroalanines at specific positions*.

Protein ID	Peptide and position of modification*	Number of modified peptides	
		Cysteine	Selenocysteine/Dehydroalanine
FdoG	LPST**X_618_**FAEENGSIVNSGR	4	4
	V**X_391_**EYIAETSAHDK	9	4
	WLSTGML**X_165_**ASASSNETGYLTQK	–	1
	VNGYI**X_554_**QGFNPVASFPNK	2	1
FdnG	WLSTGML**X_165_**ASGASNETGMLTQK	–	2
	VTGYF**X_554_**QGFNPVASFPDK	–	2
	LPST**X_618_**FAEEDGSIANSGR	2	3
	V**X_391_**EVLASTSAPDR	1	2
FdoG or	VU**_196_**HGPTVASLAPTFGR	–	8
FdnG	YTPDVVENI**X_380_**GTPK	9	9
FdoH	LIDVTT**X_39_**IGcK	7	3
	LIDVTTcIG**X_42_**K	9	2
FdnH	DEVGH**X_62_**VGVYDNPADLSAK	–	2
FdoH or	A**X_45_**QVAcSEWNDIR	2	2
FdnH	AcQVA**X_49_**SEWNDIR	1	1
	cTL**X_160_**VDR	1	1
	T**X_179_**PTGAIHFGTK	6	2
OmpA	GMGESNPVTGNT**u_311_**DNVK	1	4
	AALID**X_323_**LAPDR	7	1
NarG	GLNDVN**X_493_**ATSYDDVK	1	2
	KFSEV**X_838_**VGHLGK	–	2
	FSEV**X_838_**VGHLGK	1	2
	L**X_292_**DLWLAPK	1	2
NarH	IEAGQPTVCSET**X_263_**VGR	–	2
NarG or	KGE**X_875_**DLIPGK	–	2
NarZ	GE**X_875_**DLIPGK	–	1

Modifications confirmed by MS/MS are indicated as X, the subscript defines the respective amino acid position in the sequence.Sec-196 is the naturally occurring amino acid at position 196 and cysteine was never identified at this position (see underline peptide).

**Figure 5:**
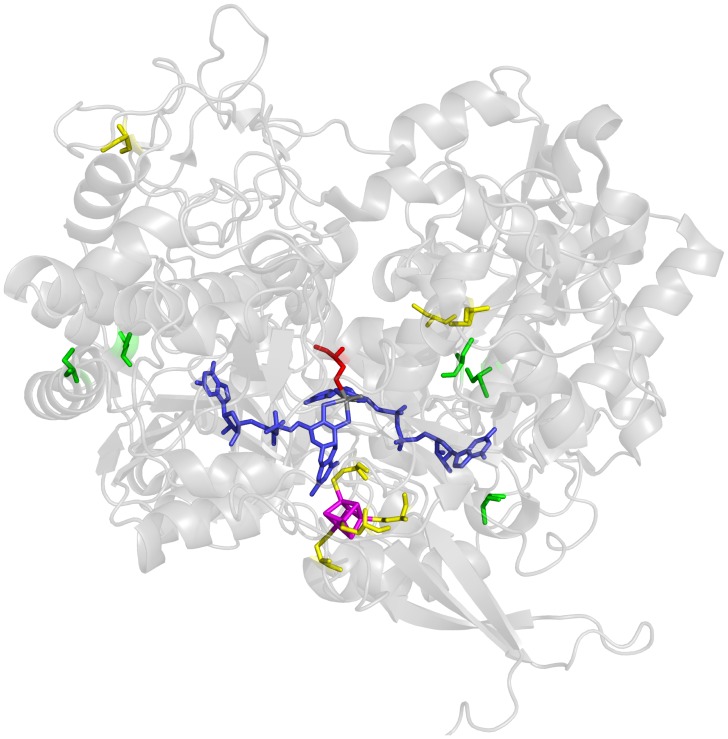
Schematic representation of the structure of the catalytic subunit FdnG of Fdh-N from ***E.*** *coli* (PDB entry: 1KQF). The protein backbone of FdnG (alpha subunit) is shown as a ribbon stucture. Modified cysteines are shown in green, unmodified cysteines in yellow. Sec-196 is presented in red. The molybdopterin guanosine dinucleotide cofactor is shown in blue and the [4Fe4S]-cluster is represented in magenta.

In both FdnG and FdoG five cysteinyl residues were identified that were never modified to selenocysteine, even after growth in the presence of 100 µM selenite (Fig. 4). Notably, three of these, Cys50, Cys53 and Cys57, together with Cys92, which could not be identified, provide the thiolate ligands that coordinate the [4Fe–4S] cluster in each catalytic subunit [18] (Fig. 5). Of the four cysteinyl residues in FdoG and three in FdnG that could not be identified, one formed part of the Tat-signal peptide and therefore was not present in the mature polypeptide analyzed here [30].

The H subunits of both Fdhs each have four [4Fe–4S] clusters and have therefore a comparatively high percentage of Cys residues [18]. Mass spectrometric analysis of both polypeptides revealed that, as with the catalytic subunit, only selected cysteines were replaced by selenocysteine (Table 2). The amino acid positions in FdnH that were substituted included Cys45, Cys49, Cys160 and Cys179, while FdoH also had substitutions in these residues as well as Cys39 and Cys42. Notably, these substitutions were in the Cys residues coordinating [4Fe–4S] clusters 1 and 4 [18]. These Cys residues were not substituted in every peptide analyzed (Table 3); however, they were all modified after cultivation of the strain at the higher selenite concentration (Table 2). The other cysteines in the polypeptide that we could identify in our MS experiments were never replaced by selenocysteine.

### Non-specific Incorporation of Selenocysteine into Other Polypeptides

NarG is a large polypeptide of 1247 amino acids and it has 12 cysteinyl residues, three of which (Cys54, Cys58 and Cys59) coordinate the [4Fe–4S] cluster in the protein [19], [20]. A paralogue of the Nar enzyme is NarZYV, which has the same overall architecture as NarGHI, is also found in *E. coli* and the NarG and NarZ share a high level of amino acid identity [31]. Our sequence coverage of NarG was approximately 60% and of the 12 cysteines in NarG, four (Cys292, Cys493, Cys838 and Cys875) were shown to be replaced by selenocysteine, but only after growth in batch culture with 2 µM selenite and not in fermenter-grown cells supplemented with 100 µM selenite (Table 2). NarG has not previously been described to contain selenocysteine.

The outer membrane protein OmpA is one of the most abundant proteins in *E. coli* [32]. The 153 amino acid protein has two cysteines. OmpA co-purified with Fdh, presumably as a contaminant (Fig. 1), but nevertheless our MS analysis revealed an approximate 55% sequence coverage of the polypeptide. After growth in the presence of 2 µM selenite, Cys323 was identified in seven peptides, while one peptide was identified with selenocysteine in place of cysteine at this position (Table 3). Amino acid 311 was identified to have a selenocysteine residue in OmpA isolated from cells grown in the presence of 100 µM selenite; however, in this instance there was a 4∶1 preference for selenocysteine over cysteine (Table 3).

We also checked the possibility that cysteines might be exchanged by other amino acids. In the proteins obtained from the purification after anaerobic growth of *E. coli* in the presence of 100 µM selenite, we identified one peptide (YTPDVVENISGTPK; *m/z* [M+H]^2+^_exp_ = 760.38570; *m/z* [M+H]^+^_theor._ = 1519.76411) from subunit G of Fdh-N and/or Fdh-O, in which Cys-380 was replaced by a seryl residue. Due to homologuous sequences in Fdh-N and -O it is impossible to assign the peptide to one or the other Fdh isoenzyme. Nevertheless, this single substitution of a cysteine for a serine in one of 20 peptides identified was the only case where an alternative amino acid was observed in the proteins studied herein. Such a substitution is highly surprising considering that the cysteyl-tRNA synthetase (CRS) displays a high level of discrimination (by a factor of 10^8^) against the misacylation of tRNA^Cys^ with serine [33], [34]. The reasons underlying this misincorporation of serine are currently unclear.

### Non-specific Incorporation of Selenocysteine into NarG is selC-Independent

Specific selenocysteine incorporation into proteins is only known to occur at certain opal (UGA) stop codons (codon 196 in *fdnG* and in *fdoG*) and in bacteria, decoding of the UGA with selenocysteine requires the *sel* gene products, in particular *selC*, specifying the tRNA^Sec^ [1], [35]. To demonstrate that substitution of cysteinyl residues with selenocysteine occurs independently of the selenocysteine-insertion machinery Nar was isolated from the membranes of the *selC* mutant FM460 grown in medium supplemented with 2 µM selenite. Translation of the *fdnG* and *fdoG* mRNA is terminated at the UGA codon 196 in the absence of selenocysteinyl-tRNA^Sec^ [3], [35]. Three exemplary selenocysteine-containing peptides of NarG are presented in Table 4, confirming the ability of the *selC* mutant to incorporate selenocysteines. Moreover, these selenocysteines were incorporated at the same positions (Cys292, Cys493 and Cys838) in Nar isolated from the wild type MC4100 (Table 2).

**Table 4:** Summary of peptides in the *E. coli selC* mutant, in which cysteines were found to be substituted by selenocysteine and converted to dehydroalanine during ESI-LTQ-Orbitrap-MS/MS.

Protein ID	Position and type of substitution^*1^	Precursor ion mass			Δm [ppm]
		*m/z* [M+H]^+^_exp_	Charge state	*m/z* [M+H]^+^_theor_	
NarG	GLNDVN**u_493_**ATSYDDVK	859.8432	+2	1718.6792	1.20
	GLNDVN**a_493_**ATSYDDVK	790.3660	+2	1579.7247	0.78
	KFSEV**u_838_**VGHLGK	704.8279	+2	1408.6486	0.06
	L**u_292_**DLWLAPK	582.2717	+2	1163.5361	0.10

Charge states of precursor ions that were selected for MS/MS analysis are indicated. Culture conditions and abbreviations are identical to those given in Table 2.

### Quantification of Selenopeptides

In order to shed light on the mechanism underlying the incorporation of selenocysteine instead of cysteine at selected positions in the polypeptides analyzed, we performed an approximate quantification of cysteines versus selenocysteines in the respective peptides (Table 3). As in the sample observed for the polypeptides isolated from cells grown with 2 µM, only dehydroalanines and no carbamidomethylated selenocysteines were present. Nevertheless, the total number of modified peptides, i.e. carbamidomethylated selenocysteine plus dehydroalanines is presented. It is immediately apparent that where cysteine-to-selenocysteine exchanges occurred, not every cysteine was exchanged at the same frequency. Our data revealed Sec/Cys ratios ranging between 0.14 and 2 (excluding Sec-196), depending on the sequence position. This ratio was independent of whether cysteine insertion was directed by an UGU or an UGC codon.

## Discussion

Selenium is an essential trace element for many organisms [2], [4]. The flipside is that excessive amounts of selenium are toxic for cells and therefore its uptake, and particularly its non-specific incorporation into proteins, must be carefully controlled. Selenite concentrations in excess of 1 µM result in non-specific incorporation of the element into *E. coli* proteins [28]. Non-specific selenium incorporation was initially proposed to occur as selenomethionine [8]; however, more recent studies provided strong evidence that selenocysteine is the main route of non-specific selenium incorporation into proteins [6], [7]. The mass spectrometric analysis presented here of two of the three selenoenzymes of *E. coli* demonstrates unequivocally not only that selenocysteine and not selenomethionine is incorporated but also that, at excessive concentrations of 2 µM and 100 µM selenite, selenocysteine replaces particular cysteines in the proteins. Initially, we chose to analyze the respiratory Fdh-N enzyme because it is an abundant selenoenzyme that can be readily isolated in its native form from the membranes of nitrate-respiring cells and because it naturally contains a single selenocysteine, which is co-translationally inserted specifically by the selenocysteine-insertion machinery [1], [17]. This selenocysteine in the protein provided an excellent internal control to validate the mass spectrometric identification of selenocysteine either as dehydroalanine [29] or as a carbamidomethylated derivative after appropriate modification.

Fortuitously, through the co-purification of the Fdh-N, Fdh-O, as well as Nar, we gleaned information on non-specific selenocysteine incorporation for all of the key components of the complete formate-nitrate respiratory pathway [16]. Despite originally being identified as a low-level formate oxidase activity in *E. coli* [12], [13] the results of the current study has confirmed a recent observation [15] that the Fdh-O enzyme has a more significant role in formate-dependent nitrate respiration than previously surmised. The large, catalytic subunits of the highly similar Fdh enzymes showed the specific, UGA-decoded selenocysteine at position 196 in their respective polypeptide, as well as the same five Cys→Sec exchanges, which are depicted in the structural representation of the FdnG polypeptide shown in Figure 5. The cysteinyl residues coordinating the [4Fe–4S] cluster were never observed to be converted into selenocysteines, regardless of the 100-fold excess in selenite added to the growth medium. While the Sec residue at amino acid position 196 was invariable and was never substituted by any other amino acid, a clear variability in the rate of substitution of the five cysteines was observed (compare Table 3). Bearing in mind the caveat that for each catalytic subunit peptides including only 10 of the 13 Cys in the case of FdnG and 10 of the 14 Cys in the case of FdoG could be identified, the fact that only five cysteines at identical positions in both proteins were substituted by selenocysteine raises the intriguing question about the mechanism that ensures non-random exchange of cysteine by selenocysteine. Furthermore, the replacement of specific cysteines by selenocysteine in the electron-transferring subunit of both Fdh enzymes, as well as in the catalytic NarG polypeptide strongly suggests that incorporation of selenocysteine at particular sites is not a random occurrence.

Examination of whether an UGU or an UGC codon specified the cysteines that were substituted or whether the relative position of the codon within the mRNA might be important did not reveal an obvious trend to explain the misincorporation of selenocysteine. Moreover, that CRS can efficiently acylate tRNA^Cys^ with either cysteine or selenocysteine is documented [6], [10] and suggests that CRS is unlikely to determine which cysteines are substituted. Finally, changing the growth temperature from 37°C to 22°C also had no influence on selenocysteine incorporation into any of the polypeptides analyzed in this study, ruling out an obvious effect of temperature on the mRNA structure. An alternative possibility is that the rate of translation of the mRNA or the codons in the vicinity of the key cysteine codons might determine whether the UGU or UGC codon is decoded with selenocysteine or cysteine. Clearly further studies will be required to elucidate the mechanism underlying this process.

The observation of misincorporation of selenocysteine at selected cysteine codons likely provides the cell with a mechanism to ‘soak-up’ excess selenium in the cell, while maintaining the functionality of the protein involved. Such a mechanism would protect thiol groups involved in coordinating key iron-sulfur clusters or catalytically important cysteines from being substituted, which would otherwise result in an inactive enzyme.

### Conclusions

In-depth mass spectrometric analyses of selenocysteine-containing Fdh-N and Fdh-O enzymes revealed the presence of both a specific, UGA-directed selenocysteine residue, as well as non-specific selenocysteines, which replaced particular cysteines in the protein. As these non-specifically incorporated selenocysteines were always found at identical positions in the protein, even in an *E. coli selC* mutant for the Nar enzyme, and no selenomethionine was identified, we suggest a mechanism must be in place for the selective replacement of particular cysteines in polypeptides with selenocysteines. This might involve selective proteolysis of catalytically inactive enzyme. The selective advantage for the cell of such a selenium-buffering mechanism would ensure maintenance of catalytic function of key enzymes, even in the presence of an excess of highly reactive selenium-containing amino acids.

## Supporting Information

Figure S1 (A) Nano-ESI-LTQ-Orbitrap-MS data. The precursor mass of the selenopeptide from FdoH with *m/z* 664.2949 matches the expected mass with a deviation of 0.26 ppm. The inset shows the characteristic isotope pattern of a selenopeptide. Signals corresponding to the selenopeptide are labeled with asterisks. (B, C) Nano-ESI-LTQ-Orbitrap-MS/MS data of the precursor ions LIDVTTcIGu_42_K (B, *m/z*664.2949) and LIDVTTcIGc_42_K (C, *m/z* 566.3073). Precursor ions were selected, fragmented, and analyzed in the linear ion trap (LTQ). MS/MS data unambiguously identify selenocysteine at position 42 of FdoH.
(DOCX)
