# Supplementary material for: Selective selC-Independent Selenocysteine Incorporation into Formate Dehydrogenases
Source: PLoS One. 2013 Apr 25;8(4):e61913. doi: 10.1371/journal.pone.0061913 (PMC3636253; doi:10.1371/journal.pone.0061913)
Supplement: Figure S1 — (A) Nano-ESI-LTQ-Orbitrap-MS data. The precursor mass of the selenopeptide from FdoH with m/z 664.2949 matches the expected mass with a deviation of 0.26 ppm. The inset shows the characteristic isotope pattern of a selenopeptide. Signals corresponding to the selenopeptide are labeled with asterisks. (B, C) Nano-ESI-LTQ-Orbitrap-MS/MS data of the precursor ions LIDVTTcIGu42K (B, m/z664.2949) and LIDVTTcIGc42K (C, m/z 566.3073). Precursor ions were selected, fragmented, and analyzed in the linear ion trap (LTQ). MS/MS data unambiguously identify selenocysteine at position 42 of FdoH. (DOCX) [file pone.0061913.s001.docx]

**Supplementary Material**

Selective selC-Independent Selenocysteine Incorporation into Formate Dehydrogenases

**
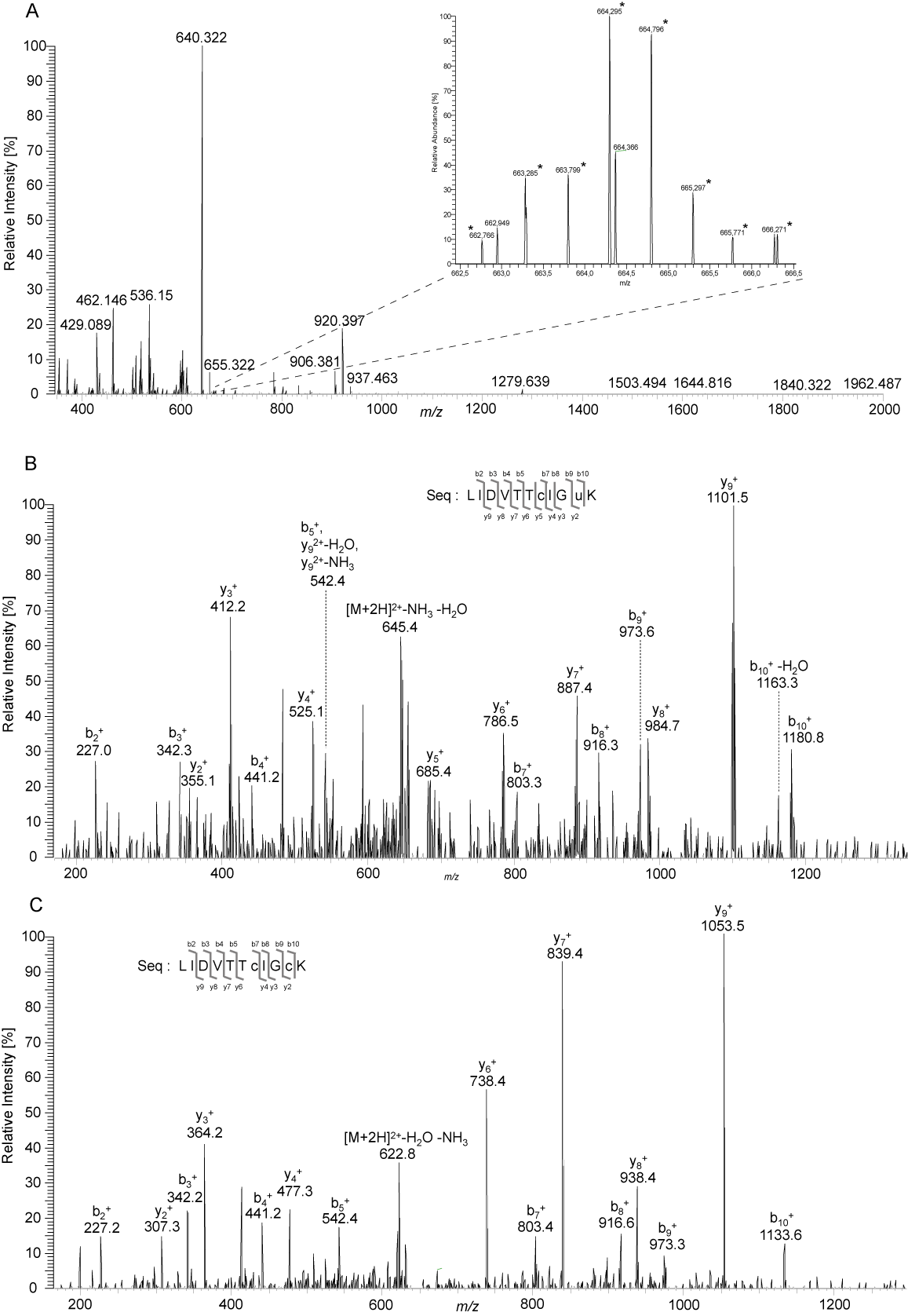
**

**Figure S1**
